# Sweden's economic inequality gap is widening and worrying

**DOI:** 10.1016/j.lanepe.2023.100610

**Published:** 2023-03-01

**Authors:** The Lancet Regional Health – Europe

Nordic countries are touted as being the most progressive, affluent, egalitarian, and aspirational. Households in the Nordic countries are among the wealthiest in the world. However, inequalities in Sweden such as income inequality and relative income poverty have increased at a faster pace than the Organisation for Economic Co-operation and Development (OECD) average in the Nordic countries over the past few decades. The Oxfam report shows that Sweden is fairing the worst in the Nordic countries at fighting economic inequality (with regard to welfare, taxes, and workers' rights). Sweden has dropped ten places in the global Commitment to Reducing Inequality Index, and has plummeted to 20th place—the lowest rank among the Nordic countries, while Norway tops the list. Sweden's Gini-coefficient—a measure of income inequality—increased sharply in 2021, reaching 0.333, which is the highest since measurements began in 1975.

Economic inequality has ripple effects on various other forms of inequalities in society, which Sweden is facing. It is a well-established fact that economic inequality leads to increase in violence, crime, poverty, and health inequalities, all of which can have lasting generational impact. Sweden is the only European country where fatal shootings have risen significantly since 2000, leaping from one of the lowest rates of gun violence on the continent to one of the highest in less than a decade. Furthermore, of the Nordic countries, Sweden has consistently had the highest at-risk-of-poverty rate since 2011: in 2020, 16% of Sweden's population lived at risk of poverty in contrast to 8.8–12.7% for other Nordic countries.

The link between socioeconomic disadvantage and poor health has been observed globally including in Sweden despite a universal tax-financed health-care provision for all residents. Although the bulk of health services in Sweden have been operated by public providers, in recent years the proportion of private (for-profit) providers has increased, particularly in outpatient care and primary health care. These reforms in Sweden might have damaged the economic equity effect of the health system. However, more detailed information at the individual level (register data) is needed to establish if the observed trends in economic inequalities really lead to economic inequalities in health.

Those at the lower end of the socioeconomic strata often have sub-standard employment that adversely effects their health. For instance, being in precarious employment with low-quality characteristics such as low income, lack of workplace rights, temporariness, and low unionisation, has been shown to increase the risk of stroke in men by 24% in Sweden. Furthermore, as per the last 2022 OECD migration report, the foreign-born population of Sweden (20.1%, 2 million) are less likely to be employed than their native-born counterparts—only 64.9% of the foreign-born population are employed, which is lower than the OECD average (69.2%). The employment rate for the native-born population is 79%, which is significantly higher than OECD average (70.01%). There is even higher inequality in unemployment rate between foreign-born (19.4%, OECD average is 9.1%) and native-born (6.8%, OECD average is 6.5%). Although Sweden has undergone large demographic changes, due to the sharp increase in pace and scale of successive migration waves in a short period of time, migration alone does not account for these high numbers of employment inequality.

This escalating trend of economic inequality is worrying for the future of Sweden's society and is largely attributed to political decisions and tax policies (favourably skewed towards high-income earners) that have not been successful in combating economic inequality. If the government does not take action to curb the escalating inequality, the future consequences can be detrimental for the country. Sweden is currently holding the Presidency of the Council of the EU from January to June, 2023. During these 6 months, the Riksdag (Sweden's parliament) will have eight conferences to discuss current EU issues in line with the EU's political agenda. Unfortunately, addressing economic inequalities is not among the four priorities to be addressed, and it is unclear whether it will be specifically addressed under the fourth priority “Democratic values and the rule of law – our foundation”.

Sweden's politics that was once known to be more tolerant is now openly more racist, with the rise in popularity of the Swedish Democratic party—the biggest force in the right-wing coalition. The Swedish Democratic party support stronger restrictions on immigration and are against providing welfare to people who are not Swedish citizens or permanent residents of Sweden, a policy known as welfare chauvinism. As the social distance between the top 10% of income earners, and the remaining 90% is growing rapidly and radically in Sweden, economic inequality that underpins all other inequalities represents a major challenge for social and public health policy and practice in Sweden. Addressing the root causes of economic inequalities by bringing reforms in tax-structure, employment system, welfare, workers' rights, and migrant-integration policies, to ensure equal conditions and opportunities for all is essential for attainting a “Wellbeing Economy” in Sweden.

